# Integration of sharp silicon nitride tips into high-speed SU8 cantilevers in a batch fabrication process

**DOI:** 10.3762/bjnano.10.226

**Published:** 2019-11-29

**Authors:** Nahid Hosseini, Matthias Neuenschwander, Oliver Peric, Santiago H Andany, Jonathan D Adams, Georg E Fantner

**Affiliations:** 1 Bioengineering department, Ecole Polytechnique Fédérale de Lausanne, EPFL STI IBI-STI LBNI, Lausanne, Switzerland; 2 Biophysik Department, ETH Zürich, Basel, Switzerland

**Keywords:** Atomic force microscopy (AFM), durability, imaging speed, polymer cantilever, silicon nitride tip

## Abstract

Employing polymer cantilevers has shown to outperform using their silicon or silicon nitride analogues concerning the imaging speed of atomic force microscopy (AFM) in tapping mode (intermittent contact mode with amplitude modulation) by up to one order of magnitude. However, tips of the cantilever made out of a polymer material do not meet the requirements for tip sharpness and durability. Combining the high imaging bandwidth of polymer cantilevers with making sharp and wear-resistant tips is essential for a future adoption of polymer cantilevers in routine AFM use. In this work, we have developed a batch fabrication process to integrate silicon nitride tips with an average tip radius of 9 ± 2 nm into high-speed SU8 cantilevers. Key aspects of the process are the mechanical anchoring of a moulded silicon nitride tip and a two-step release process. The fabrication recipe can be adjusted to any photo-processable polymer cantilever.

## Introduction

Atomic force microscopy (AFM) cantilevers have been developed for numerous applications since the invention of scanning probe microscopy (SPM) [1]. Quality and accuracy of an AFM image strongly depend on the tip geometry since the image topography is the convolution of the surface topography and the cantilever tip geometry [2]. More precisely, the resulting images suffer from the effect of dilation [3]. AFM images with tip artefacts are of reduced quality and can seriously mislead users [4]. New fabrication methods have enabled increased tip sharpness and uniformity, so that commercial AFM cantilevers now have a standard tip quality. A range of specialized AFM techniques require custom tip designs, including high-speed AFM [5–6], high-resolution electrochemical and nanoelectrical imaging [7–8], Raman spectroscopy [9], nanoindentation [10], nanomechanical machining [11], plasmonic applications [12–13] and microscale grapping [14].

In parallel with the development of AFM cantilevers made out of traditional materials (e.g., silicon, silicon nitride and silicon oxide), polymer cantilevers have gained attention due to their ease of fabrication, their versatility [15–19] and their potential for fabricating low spring constant cantilevers [20]. For instance, the microfabrication process of SU8 cantilevers has a high fabrication yield and an easy bottom-up recipe. Genolet et al. have shown AFM images of DNA-plasmid molecules using SU8 cantilevers [21]. SU8-based Hall effect sensor cantilevers have also been presented by Mouaziz and co-workers [22].

In addition, SU8 cantilevers have shown a performance of high-speed amplitude modulation AFM (HS-AM-AFM) enhanced by up to one order of magnitude due to their low mechanical quality factor (Q-factor) and hence their high mechanical bandwidth [23]. A tip made of SU8 or other structural polymers can be integrated into a polymer cantilever by moulding. Such tips have been prepared with acceptable radii for many imaging purposes [20]. However, the wear rate of SU8 is very high [24], which makes this and other polymers a nonideal tip material. Some attempts to coat SU8 cantilevers and tips with a more wear-resistant material (such as graphene) have been made [25], but yielded blunt tips (the tip radius increased by an order of magnitude).

Lee et al. have shown that hydrogel AFM cantilevers fabricated by replica moulding and UV curing have great potential for tuning the mechanical properties of the tip, its shape and the surface functionalization [26]. However, the fabrication of hydrogel probes requires processes that involve individual alignment and bonding [27].

The present work aims to overcome the primary limitation of polymer AFM cantilevers, namely the poor wear rate of polymer tips, by integrating a tip element made of a traditional tip material. The main concept of this work is to partially embed the tip into the cantilever body, such that the attachment between the tip and the polymer is of a mechanical nature. We have developed a batch fabrication process to integrate silicon nitride tips into SU8 cantilevers. The whole structure, except for the tip, is made of SU8 to benefit from the ease of fabrication and the high-speed imaging capability of cantilevers made of this polymer, while oxide-sharpened silicon nitride tips provide tip sharpness and tip wear-resistance. The tip is anchored securely by being partially embedded in the polymer cantilever. These probes therefore have the advantage of a fast mechanical response, and the tip is made from a material that is known and accepted in the field as suitable for high-quality tips.

## Cantilever Fabrication

**Figure 1:**
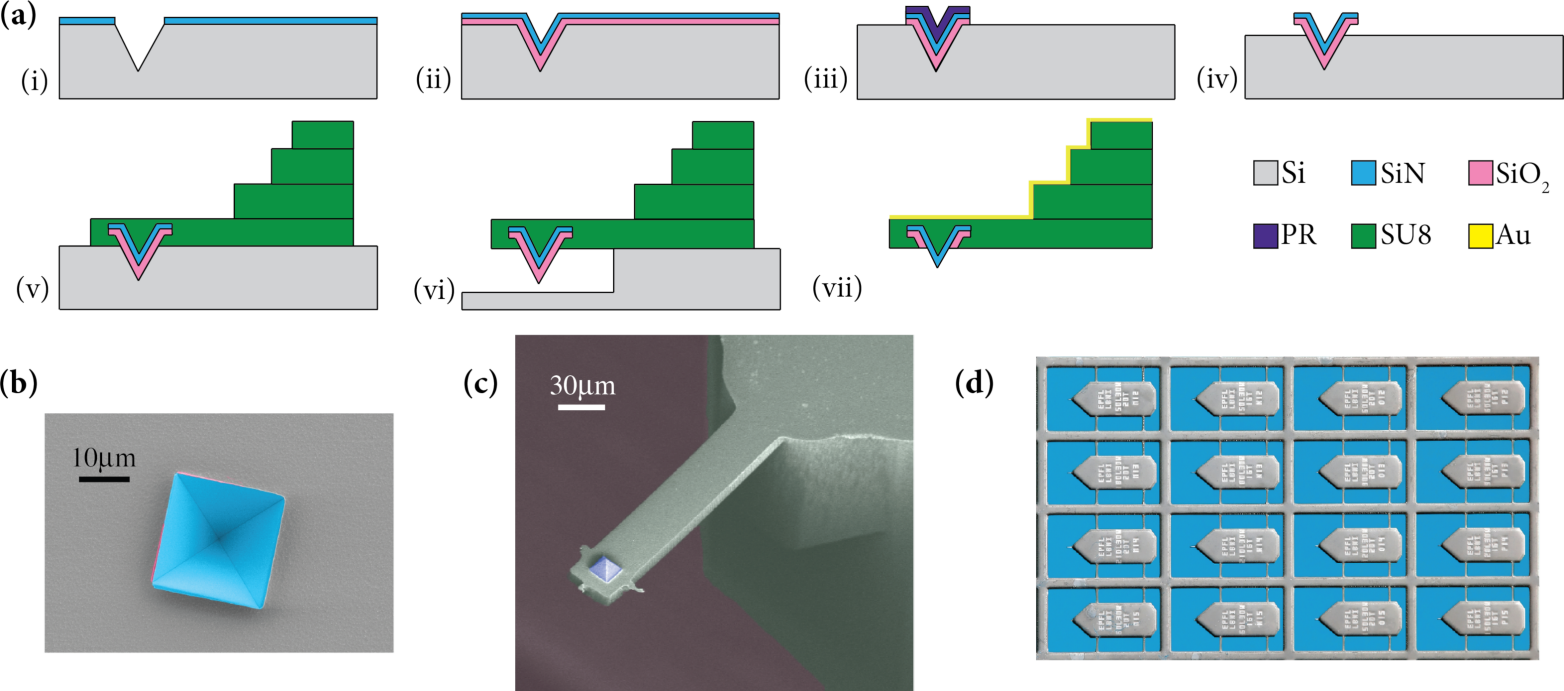
Batch fabrication process of LSNT-tip SU8 cantilevers. (a) Summarized process flow. (b) SEM image of the LSNT and the silicon oxide layers where the silicon underneath has been etched (step iv). (c) SEM image of a single cantilever. The pyramidal tip consists of four {111}-planes and has a half-cone angle of 35°. It is aligned with the cantilever. (d) Optical photograph of the released cantilevers.

The cantilever is made of SU8 and the tip is entirely covered with low-stress silicon nitride (LSNT). Pyramidal tips are made based on an indirect tip fabrication process [28] by etching a mould into a 380 µm thick single-side polished silicon (100) wafer. Figure 1a shows the summarized process flow, outlining the important steps.

(i) A 20 nm LSNT thin film is layered onto a silicon (100) wafer by low-pressure chemical vapor deposition. Circular openings (20 µm diameter) are then cut into the layer by electron-beam lithography. The LSNT mask is dry-etched before the moulds are structured by anisotropic KOH (40% at 60 °C) etching. The formation of {111} facets results in four-sided pyramidal pits. The diameter of the circular openings defines the final height of the tips and can be tuned. (ii) The LSNT mask is removed in HF 50%. Afterwards, a 400 nm wet silicon oxide layer and a 100 nm LSNT layer are deposited on the wafer. The 400 nm silicon oxide layer improves the tip sharpness by oxidation sharpening [29]. Studies report a 30% decrease of the oxide thickness along the sharp silicon ridges after wet oxidation at 900–950 °C [30]. This decrease is due to an increase of the activation barrier of the interfacial reactions induced by the stress build-up in these areas [31]. Due to the nonlinear growth of the silicon oxide, the oxide layer becomes thinner at the inside corner of the pyramidal moulds than at the mould faces. The silicon oxide layer forms a concave curvature on each face of the four-sided pyramidal moulds, which is then projected onto the subsequent LSNT layer. (iii) The silicon oxide and the LSNT layers are patterned by photolithography to cover only the etched pits. (iv) Deep reactive ion etching (DRIE) is used to etch the silicon vertically and laterally (4 and 1 µm, respectively) in order to provide access for the SU8 polymer to fill the base of the tips in the subsequent steps. Figure 1b shows the SEM image of this step. (v) All SU8 (GM1050, GM1060 and GM1075, Gersteltec, Pully, Switzerland) structural layers, the cantilever beam and the three layers of the chip body are patterned by photolithography. A three-layer chip body with an offset between the successive layers is required especially for shorter cantilevers, so that the chip body does not obstruct the path of the laser for the optical readout. The thicknesses of the chip body layers are, from bottom to top, 30, 120 and 150 µm. The geometry of the SU8 beam defines the resonance frequency of the cantilever (*f*_0_) and the spring constant (*k*). (vi) The process is designed for top release, so the wafer is treated with DRIE to create a freestanding SU8 beam with the embedded silicon nitride tip encased in a protective oxide. (vii) The release process is finalized by placing the wafer in KOH (23% at 90 °C) to separate the SU8 cantilevers from the wafer. The silicon oxide layer on the tips is then stripped using buffered hydrofluoric acid. The process is completed by titanium–gold (5–20 nm) sputtering on the chip-body side of the cantilevers. This layer serves as the reflective metal coating required for the optical beam deflection read out. Figure 1c and Figure 1d show the SEM and the optical images of the released cantilevers fabricated by this process.

## Results

The primary goals of the fabrication of AFM cantilevers for general imaging purposes are to enhance the tip sharpness, improve the tip durability and to increase the detection speed and sensitivity. The detection speed in amplitude-modulation mode is determined by the amplitude response time of the cantilever. The tapping-mode bandwidth is given by BW *= πf_0_/Q*, where *f*_0_ is the resonance frequency and *Q* is the Q-factor [32]. The resonance frequency for a rectangular cantilever with homogenous material properties and no external load is given by


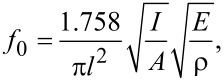


where *E* is the elastic modulus, *I* the second moment of area, ρ the density and *A* the cross-sectional area of the cantilever beam. Thus, the resonance frequency depends on the properties of the cantilever material, which are presented as 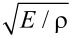. On the other hand, 1/*Q* is strongly influenced by the intrinsic damping 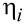 of the cantilever material. Therefore, optimizing the ratio π*f*_0_*/Q* translates into optimizing the ratio 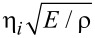, which has been defined as material bandwidth product [23]. SU8 cantilevers have shown a high imaging speed due to the high material bandwidth product, which mainly results from the high intrinsic damping properties of the polymer. Such cantilevers have high resonance frequencies and low Q-factors for a given size and stiffness [23]. However, SU8 tips wear down quickly and become blunt when they encounter hard surfaces with high aspect ratio features [24]. The SU8 cantilever fabrication process that we have developed has overcome this issue by incorporating hard LSNT-tips into the process without sacrificing the high detection bandwidth of the polymer levers.

To quantify the tip sharpness, 20 randomly chosen LSNT-tip SU8 cantilevers have been tested with a polycrystalline titanium roughness sample. The images were taken using a NanoScope-V controller and a Multi-Mode-V AFM with a J scanner (Bruker) in tapping mode. The imaging conditions were as follows: scan size 2 µm, number of pixels 512 × 512, scan rate 1 Hz, free amplitude 123 nm and setpoint at 95% of the free amplitude.

**Figure 2:**
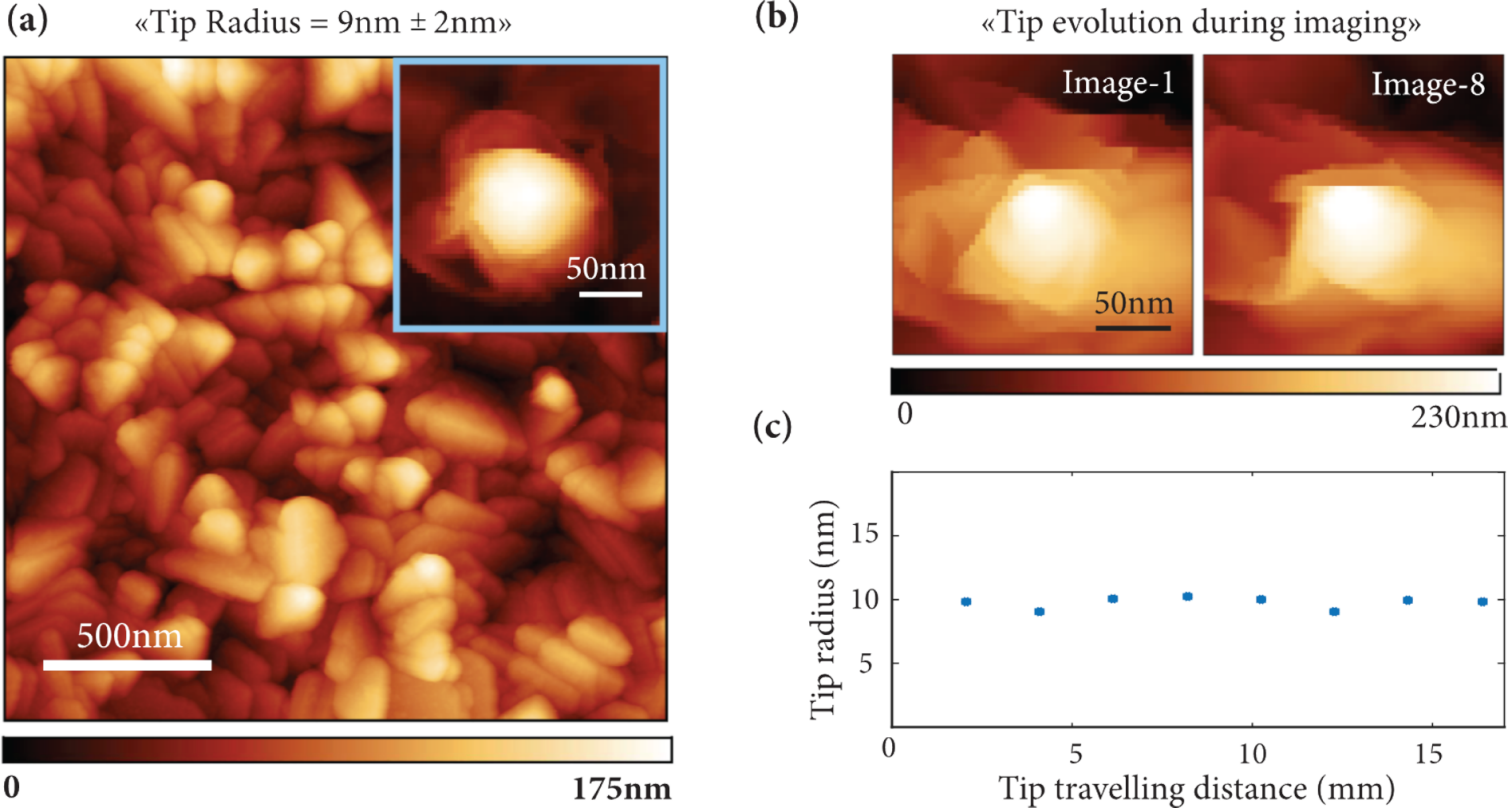
Tip sharpness and durability assessment of the LSNT-tip SU8 cantilevers using a polycrystalline titanium roughness sample in tapping mode. (a) The AFM image of the polycrystalline titanium roughness sample, used for the tip radius measurement. The average tip radius of 20 randomly chosen cantilevers is 9 nm. The image size is 2 × 2 µm at 512 × 512 pixels while the array dimension for the tip estimation is 55 × 55 pixels. The inset shows the result of the Gwyddion partial tip blind estimation extracted from the full image. (b, c) the tip radius evolution over 16 mm travelling distance shows negligible tip degradation.

With these parameters, we estimated the tip–sample forces using the Virtual Environment for Dynamic AFM (VEDA, nanohub.org/tools/veda) and obtained mean forces of 10 nN. Figure 2a shows an AFM image taken with one of the LSNT-tip SU8 cantilevers.

To evaluate the tip sharpness, the blind tip estimation algorithm [33] as implemented in the Gwyddion program [34–35] has been used. The blind tip estimation algorithm is used to estimate the sharpness of the tip from the image of a polycrystalline titanium tip characterizer sample of unknown geometry, with features significantly sharper than the tip under evaluation. The Gwyddion partial blind tip estimation algorithm iterates over the surface of the image to find the high points with the steepest slopes on the image. These points are subsequently used to estimate the radius of the tip 5 nm away from its apex by taking the average width of the tip along the two orthogonal axes, using the assumption that the evaluated tip must be sharper than the sharpest feature on the image of the specimen. To guarantee that the dilation of the specimen surface results exclusively from the tip geometry, the noise suppression threshold is set at 100 pm which is superior to the measured image noise of 40 pm. Additionally, borders of the image are also excluded from the estimation to prevent edge artefacts. The inset of Figure 2a shows the result of the Gwyddion partial tip estimation, which uses a limited number of the highest points on the image to estimate the sharpness of the tip. For the 20 cantilevers we evaluated, we obtained an average tip radius of 9 nm with a standard deviation of 2 nm.

We evaluated the tip durability in our work by uninterrupted imaging of the polycrystalline titanium roughness sample (tapping mode, scan size 2 µm, 512 × 512 pixels and scan rate 1 Hz). Figure 2b shows the partial blind estimation of the tip shape for the first and last images after 16 mm tip travel. No obvious degradation occurred. Figure 2c shows the evolution of the tip radius for more than 16 mm of tip travel (8 images).

**Figure 3:**
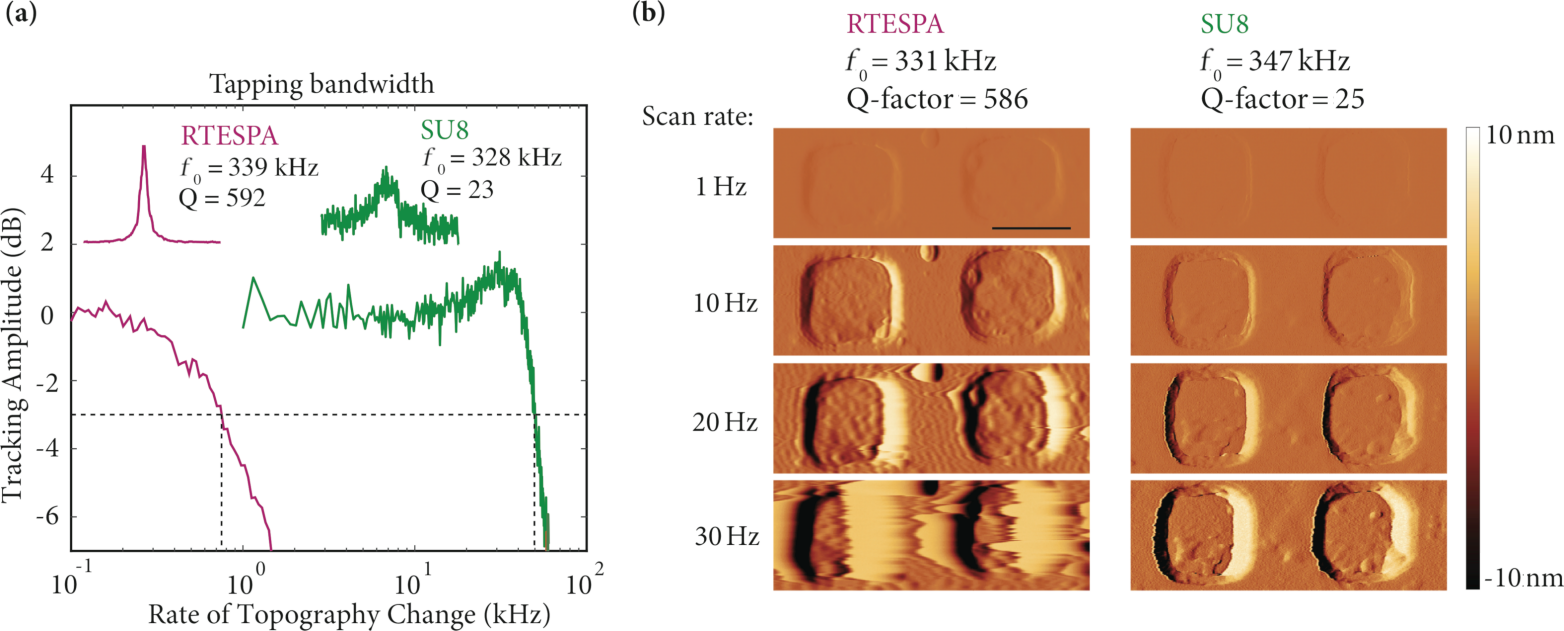
Comparison of the tapping bandwidth between our tip-integrated SU8 cantilever and a commercial silicon cantilever (RTESPA). (a) The 3 dB drop of the surface tracking in tapping mode for RTESPA and the SU8 cantilever happens at 750 Hz and 50 kHz, respectively. RTESPA has *f*_0_ = 339 kHz and *Q* = 592, and the LSNT-tip SU8 cantilever has *f*_0_ = 328 kHz and *Q* = 23. (b) Amplitude error images of a 1 µm pitch reference sample taken by the RTESPA silicon cantilever and the LSNT-tip SU8 cantilever at scan rates of 1, 10, 20 and 30 Hz. The SU8 cantilever shows a better topography tracking ability compared to the RTESPA cantilever due to its higher tapping bandwidth. The scale bar is 500 nm.

To investigate the detection speed of the LSNT-tip SU8 cantilevers, we measured the detection bandwidth of the cantilever in tapping mode by measuring the 3 dB decrease of the tracking amplitude similar to the protocols described by Kokavecz et al. and Sulcheck and co-workers [36–37]. Figure 3a shows a comparison of the bandwidths of the individual cantilevers, namely the commercial RTESPA (Bruker AFM probes, Camarillo, CA, USA) with *f*_0_ = 339 kHz, *k* = 48 N/m, *Q* = 592, planar dimensions of 125 × 40 µm and a thickness of 3.4 µm, and our LSNT-tip SU8 cantilever with *f*_0_ = 328 kHz, *k* = 15 N/m, *Q* = 23, planar dimensions of 80 × 20 µm and a thickness of 7 µm. The detection bandwidth is 750 Hz and 50 kHz for the RTESPA and SU8 cantilevers, respectively.

Both cantilevers were designed for tapping-mode AFM imaging in air. We want to point out that based on our choice of planar geometries and thickness of the cantilevers, these two cantilevers were the most similar in terms of resonance frequency, however, their parameters are not an ideal match. Nevertheless, the drastically higher bandwidth of the SU8 cantilever is to be primarily attributed to the change in the materials properties. The LSNT-tip SU8 cantilever is more than 50 times faster than its silicon cantilever counterpart for a given resonance frequency. In order to evaluate the link between the tapping bandwidth and the image quality, an AFM calibration grating (1 µm pitch, 100 nm depth) was imaged with the RTESPA cantilever (*f*_0_ = 331 kHz, *Q* = 586) and the LSNT-tip SU8 cantilever (*f*_0_ = 347 kHz, *Q* = 25) at scan rates of 1, 10, 20 and 30 Hz. The imaging was conducted with a Bruker Dimension FastScan AFM system at a scan size of 2 µm and a number of pixels of 512 × 512. Figure 3b shows the amplitude error images taken at different scan rates for these two cantilevers. A lower amplitude error contrast corresponds to a better tracking performance. While the silicon cantilever clearly tracks the sample poorly at a scan rate of 30 Hz, the SU8 cantilever detects the sample topography significantly better.

## Discussion

The critical feature of any AFM cantilever is the tip. For general imaging, the quality of the tip is primarily determined by the tip radius and the wear rate of the tip. We need to comment that our tips have a decent sharpness compared to other silicon nitride moulded tips, but they are less sharp than the typical silicon tips that are commercially available with tip radii of less than 2 nm. However, even for the ultra-sharp tips, tip wear is unavoidable and a great concern for AFM users. The tip wear problem has been reported as early as 1991 [38]. By assuming that all of the imaging parameters are set correctly, tip wear can still occur due to abrasive wear, fracture and adhesive wear [39–40]. The presented cantilevers have proven a good tip sharpness, and provide the well-established wear-resistance of silicon nitride tips, supplemented with their good tracking bandwidth. We should also mention that our tips have a relatively large opening angle of 35°. On the one hand, the large opening angle limits the imaging capabilities on very rough samples. On the other hand, our tips are symmetric and have a clearly defined geometry, which can be beneficial, for example for nanomechanical mapping of biological samples.

In general, SU8 cantilevers suffer from residual mean stress and residual stress gradients in the beam. These residual stresses can bend the cantilevers and cause issues with aligning the laser and approaching the sample. Keller et al. have shown that introducing a long hard bake after the SU8 development, and a modification of the SU8 photolithography baking profiles make it possible to fabricate 500 µm long cantilevers with less than 20 µm initial bending for 2 µm thick SU8 cantilevers [41]. Although our cantilevers are already relatively straight (due to their shorter length), a similar optimization of the process parameters could improve this issue further.

The cantilevers shown in Figure 1c have a peculiar shape with SU8 residues sticking out in a cross at the free end of the SU8 cantilever. This is due to the scattering of the light during photolithography of the cantilever patterning. Light travels through the SU8 polymer and reaches the bottom surface (LSNT) and is then reflected back to the parts of the non-exposed SU8. Except for its unusual shape, it does not bring any prominent problem in general AFM uses unless the cross is so large that it touches the sample, which we have not observed so far. One way to reduce this problem could be adjusting the exposure dose to values not higher than absolutely required.

The increased detection bandwidth of the SU8 cantilevers arises from the viscoelastic nature of the SU8 polymer resulting in a low Q-factor. This low Q-factor however comes at the price of a low mechanical excitation efficiency when shaking the cantilever at resonance with a dither piezo. Hence the drive amplitude for these cantilevers has to be higher than that of traditional cantilevers. This gives rise to parasitic resonance peaks in the cantilever tune, which is well known for tapping-mode AFM in low-*Q* environments such as liquids. As with imaging in fluids, acquiring a thermal tune prior to the mechanical tune helps to find the correct resonance peak to use. The poor mechanical tune caused by the low Q-factor of the cantilever is aggravated by the fact that the chip body is also made of SU8 instead of a stiff conventional material. One technique to approach this challenge would be to make the cantilever chip body out of a SU8 nanocomposite with higher Young’s modulus instead of pure SU8. For instance, M. Kandpal et al. [42] have shown that embedding ZnO nanoparticles into a pure SU8 matrix increases its Young’s modulus from 8 to 30 GPa. The stiffer cantilever chip body will probably yield better mechanical tuning properties and hence an improved ease of use.

## Conclusion

In this article, a batch fabrication process of LSNT-tip SU8 cantilevers has been presented. Tip sharpness measurements have been performed for 20 cantilevers, and reveal an average tip sharpness of 9 ± 2 nm. The tips are made of LSNT, a material known for its wear resistance, and no clear wear was observed after more than 16 mm of tip travel during the AFM imaging of a polycrystalline titanium roughness sample.

A suitable tip sharpness and a high wear resistance have been achieved along with a high tracking bandwidth of the fabricated LSNT-tip SU8 cantilevers. A comparison between a commercial silicon cantilever and the LSNT-tip SU8 cantilever reveals that the detection speed is improved by a factor of more than 50.
